# Latent Factors in Attention Emerge from 9 Years of Age among Elementary School Children

**DOI:** 10.3389/fpsyg.2017.01725

**Published:** 2017-10-05

**Authors:** Ting Tao, Ligang Wang, Chunlei Fan, Wenbin Gao, Jiannong Shi

**Affiliations:** ^1^Key Laboratory of Mental Health, Institute of Psychology, Chinese Academy of Sciences, Beijing, China; ^2^Key Laboratory of Behavioral Science, Institute of Psychology, Chinese Academy of Sciences, Beijing, China

**Keywords:** elementary school children, perceptual attention, executive attention, development, factor analysis

## Abstract

We explored the development of attention among elementary school children. Three hundred and sixty-five primary school children aged 7–12 years completed seven attention tests (alertness, focused attention, divided attention, attentional switching, sustained attention, spatial attention, and supervisory attention). A factor analysis indicated that there was no stable construct of attention among 7- to 8-year-old children. However, from 9 years on, children’s attention could be separated into perceptual and executive attention. Notably, however, the attention types included in these two factors differed from those among adults.

## Introduction

Attention remains a highly popular topic in cognitive psychology, and researchers have conceived a multitude of concepts and related measures of attention. A number of currently popular attention models can be traced back to the work of Posner and Petersen (1990), who proposed that the attentional network can be divided into three parts: alerting, orienting, and executive control (Posner and Boies, 1971; Fan et al., 2002). Coull (1998) made a similar classification of attention based on neuropsychology research. Sturm and Zimmermann (2000) distinguished between the various types of attention based on Posner’s work, including alertness, focused/selective attention, spatial attention, attentional switching, sustained attention, and divided attention. In sum, while researchers have widely differing understandings of the concept of attention, there is one common view: namely, that it is a multidimensional construct comprising several subcomponents or functions (Mesulam, 1981; Mirsky et al., 1991; Fan et al., 2002; Posner, 2012). However, the internal structure of this construct has received considerably less attention. Accordingly, some researchers have begun to consider the possibility of theoretically integrating different types of attention. At present, there are several theories backed by empirical research.

The theory of visual attention proposed by Bundesen (1990) assumes that perceptual categorization forms the foundation of both visual recognition and selection of objects. The process of recognition/selection of an object indicates that the object is assigned to one of several alternative categories. In this context, attentional recognition/selection is assumed to be a choice process, which implies a race between other alternative perceptual categorizations. Finally, the first categorization to complete wins, and the object is categorized. The time required for completing the categorization process depends on various parameters, such as the strength of sensory evidence supporting a categorization, the attentional weight of an object, and the subject’s bias for assigning an object to a particular category. Drawing on the theory of visual attention developed by Bundesen (1990) and Logan and Gordon (2001) proposed a theory of executive control wherein they claim that it is composed of two controllable subordinate processes. The first is *stimulus selection*, taken directly from the theory of visual attention, and the other is *response selection*, which was adapted from exemplar-based random walk model (Nosofsky and Palmeri, 1997). Furthermore, while Bundesen (1990) focused on stimulus selection within tasks, Logan and Gordon (2001) emphasized the selection process between tasks. More specifically, in Logan and Gordon’s theory, each task set includes various control parameters (e.g., attentional weight, decision bias) that are necessary for programming attentional selection to perform a particular task. Initially, the task sets are represented in working memory. Then, the control parameters within these task sets are transferred from working memory to the subordinate processes, as suggested by the theory of visual attention and exemplar-based random walk models. Numerous dual-task phenomena, such as crosstalk, set-switching costs, and concurrence costs, can be explained by Logan and Gordon’s executive control theory – in particular, they claim that these effects are due to the theory of visual attention processes being run twice – once on Stimulus 1 and once on Stimulus 2.

Following theoretical considerations that relate attention to perception (Bundesen, 1990) and also to the executive control of performance in complex tasks (Logan and Gordon, 2001), two latent factors underlying individual differences in attention measures are assumed: perceptual attention and executive attention. Some empirical studies on adults support this hypothesis: Based on studies by Sturm and Zimmermann (2000), Schweizer et al. (2005) proposed that attention can be divided into three levels from the perspective of mental processing: perceptual attention, higher mental attention, and executive attention. They tested adults’ attention using 14 attention tasks. The results showed that attention can be divided into two latent factors. One is perceptual attention. Higher mental attention and executive attention were integrated into one factor since there were too many common factors in them, and it was named executive attention (Schweizer et al., 2005). In their subsequent study, the two-factor structure was also confirmed. They further classified alertness, focused attention, sustained attention, and spatial attention as perceptual attention, while attentional switching, divided attention, and supervisory attention were classified as executive attention (Moosbrugger et al., 2006).

Previous studies on attention structure have largely concentrated on adults. This begs the question of whether this dichotomy exists in children. Furthermore, if it exists, at what age does it appear? Exploring the development of this attention structure in children would not only clarify our understanding of the structure of this basic cognitive ability but also its role in cognitive development. However, adult modes cannot explain transitions from one stage to the next in child development (Arsalidou and Pascual-Leone, 2016). From the perspective of constructivist developmental theories, age 7–12 years is a concrete operation stage (Piaget, 1964). The stage before this one is pre-operations and the stage after this one is formal operations. As the transitory stage from pre-operations to formal operations, this stage is relatively long. However, children’s basic cognitive abilities do not keep stable in this stage, they develop subtly. In Pascual-Leone and Baillargeon (1994) work of measuring mental attention, this stage was divided into three sub-stages: 7–8 years is defined as low concrete operations, 9–10 years is defined as high concrete operations, and 11–12 years is defined as transition to formal operations. What’s more, Duan’s (2009) study on children’s executive function found that there was no stable executive function construct in 7- to 8-year-olds but a stable construct similar to the one observed in adults was found among 9- to 12-year-olds. Based on these, it is hypothesized that attention can be separated into perceptual and executive attention from the age of 9 years onward. Drawing on the classification of attention types suggested by Sturm and Zimmermann (2000) and the supervisory component of attention, we probed the developmental trajectory of attention structure in elementary school children using seven attention tests (alertness, focused attention, divided attention, attentional switching, sustained attention, spatial attention, and supervisory attention).

## Materials and Methods

### Participants

Participants (*N* = 365) were 104 7- to 8-year-olds (*M* = 7.93, *SD* = 0.36), 142 9- to 10-year-olds (*M* = 9.57, *SD* = 0.60), and 119 11- to 12-year-olds (*M* = 11.39, *SD* = 0.49) from a public elementary school in Beijing. The participants had an approximately even gender ratio (46.58% female), and were from a middle-income school. Only children who received parental consent and provided their own assent completed the protocol. All participants had normal or corrected-to-normal vision. Participants’ demographic details are shown in **Table 1**.

**Table 1:** Demographic characteristics of the sample.

Age groups	7–8 (*n* = 104)	9–10 (*n* = 142)	11–12 (*n* = 119)
Mean ±*SD*	7.93 ± 0.36	9.57 ± 0.60	11.39 ± 0.49
Gender (M/F)	56/48	77/65	62/57

### Materials and Procedure

Participants completed seven tests, which assessed their alertness, focused attention, divided attention, attentional switching, sustained attention, spatial attention, and supervisory attention. Among these, the tests of alertness, focused attention, divided attention, and attentional switching were sourced from the Test for Attentional Performance (Zimmermann and Fimm, 2000). Sustained attention was measured using the Continuous Performance Task (Conners et al., 2003), while spatial attention was assessed with the spatial attention test from the Multidimensional Attention Test battery (Schweizer et al., 2005). Finally, the Star Counting Test was used to test supervisory attention (de Jong and Das-Smaal, 1990). The reason that we chose these particular tests, in addition to being adequate measures of these concepts, is that they were readily available.

#### Alertness

Alertness was assessed using a reaction time task, whereby participants were asked to respond to the appearance of a large X in the middle of a computer screen by pressing the response key as fast as possible. Stimuli were presented in the center of the screen with a visual angle of approximately 1.1° vertical and 1.1° horizontal. Upon their response or no longer than 2,000 ms after the initial presentation, the target disappears from the screen. The interstimulus interval (ISI) varied randomly between 500 and 2,000 ms, and in some trials a tone preceded the target, with the temporal distance between the tone and target varying from 166 to 1,000 ms. The task comprised 80 trials; in the first and last 20 of these trials, the target was presented without a tone, while in the middle 40 trials, the target was presented in combination with a tone. The time between the presentation of the target and the response is measured and stored.

#### Focused Attention

This task requires the identification of targets, which comprise five stimuli, each of a regular texture that makes them easily discernable from each other, all of which are presented inside a square. Stimuli were presented in the center of the screen with a visual angle of approximately 1.4° vertical and 1.9° horizontal. Two of the five stimuli were the targets. After the appearance of either, participants had to press the response key as quickly as possible. When a non-target appeared, however, they had to abstain from a response. The stimulus was presented until the participant pressed the key. In case of no reaction, the stimulus would remain on the screen for 1,000 ms. The ISI varied randomly from 1,000 to 2,000 ms. Of the 60 trials, 24 required a response. The time between the presentation of the target and the response was measured and stored.

#### Sustained Attention

Sustained attention was measured using a typical continuous performance task, during which 540 numbers (approximately 2 cm in size) appeared in the middle of a computer screen, one at a time, for 200 ms. The ISI was 1,000 ms. Stimuli were presented in the center of the screen with a visual angle of approximately 2.6° vertical and 1.8° horizontal. Participants were asked to press the response key if the number 9 was preceded by the number 1; the event rate was 10%. The time between the presentation of the target and the response was measured and stored.

#### Divided Attention

Divided attention was measured using a dual-task paradigm comprising visual and acoustic components. In the visual task, several small Xs and dots appeared at random positions within a 4 × 4 reticule in the middle of the computer screen for 2,000 ms. Stimuli were presented in the center of the screen with a visual angle of approximately 3.4° vertical and 4.4° horizontal. The participant was asked to press the response key when a set of four Xs formed the corners of a square. There was no ISI between the trials. In the acoustic task, a sequence of high and low beeps was played to the participant, and they had to respond as quickly as possible whenever a sequence of two low or two high beeps was presented. The same response key was used for both tasks. The time between the presentation of the (visual or acoustic) target and the response was measured and stored. There were 100 visual trials and 200 auditory beeps; spread across these trials, there were 17 visual targets and 16 auditory targets.

#### Spatial Attention

This task measures the quickness of access to external information appearing at various locations. In each trial, a stimulus consisting of a small cross and four circles inside a square was presented to participants. The cross is located in the middle of the computer screen and each circle is placed near one of the four corners of the square. Stimuli were presented in the center of the screen with a visual angle of approximately 5.1° vertical and 5.1° horizontal. After a break of 500 ms, an arrow directed toward one of the circles replaced the cross for 1,000 to 1,500 ms until a small dot – the target – appeared within one of the four circles. A total of 120 trials were held. In 80% of the trials, the target appeared in the circle that the arrow pointed to. The ISI was 500 ms and there were 120 trials. The participant was instructed to keep their eyes fixed at the center of the screen, to move their attention in the direction of the arrow, and to respond as quickly as possible to the appearance of the target. Stimulus was presented until the participants pressed the key. The time between the presentation of the target and the response was measured and stored.

#### Attentional Switching

In this task, a letter and a number appeared to the left and right of the center of the computer screen; their positions changed randomly throughout the experiment. Stimuli were presented in the center of the screen with a visual angle of approximately 0.7° vertical and 6.0° horizontal. The presentation time of the stimuli depended on participants’ responses, but the ISI was always 150 ms. In the first trial (out of 100 total), the participant was asked to determine whether the letter was located on the left or right and to press the response key corresponding to that location. In the next trial, the participant was asked to indicate the location of the number. The target thus alternated with each trial (e.g., the third trial was like the first trial and the fourth trial was like the second). Stimulus was presented until the participants pressed the key. The time between the presentation of the target and the response is measured and stored.

#### Supervisory Attention

The 20-trial computerized version of the Star Counting Test comprises a variety of stimuli, including arrays of stars, some “+” and “-” signs, and a starting number presented on the left side of the stars. Stimuli were presented in the center of the screen. As the size of the stimuli changed along with the numbers of stars in each trial, the visual angle is not reported here. The participant was asked to add one to the starting number for each star that appeared as they scanned the array from left to right. When they encountered a “-” sign, they had to subtract 1 from the running total until encountering the next “+” sign. The participant was instructed to scan the array as fast as possible and to press the response key immediately after completing the additions and subtractions. Afterward, the material was removed and the participant was prompted to enter the result of their counting into the computer. The time required for counting (only the correct trials) was used as a measure of efficiency. Various sources have supported the validity of the computerized version of this test (Schweizer and Koch, 2003).

All the tasks were programmed with E-prime 1.1 Software. The test room was illuminated normally. All the trials were presented in task designs. The tests were administered over two 45-min sessions on different days (separated by a maximum of 1 week) in order to avoid exhausting participants. Furthermore, they were provided with a rest after every task. To make sure that participants understood the task requirements, we divided them into groups of four to six children before the experimenter gave a detailed introduction of each task. Then, participants engaged in several practice trials before beginning the formal task. During practice trials, the experimenters observed the participants’ responses to confirm whether they understood the task requirements. If the experimenter found that the participants did not understand the task requirements and made numerous errors, they repeated the detailed introduction of the task to participants and gave them another opportunity to practice. Each child was not allowed to proceed to the formal experiment until the experimenter was confident that they completely understood the task. Two graduate students majoring in psychology who had been trained in conducting the experiment collected all experimental data.

Before participation, all parents and children signed informed consent forms. The study was approved by the Ethics Committee of the Institute of Psychology, Chinese Academy of Sciences.

## Results

### Descriptive Results

The accuracy results for the seven attention tests are presented in **Table 2**. In order to compare the effects of age group differences on accuracy of the attention tasks, we performed seven analyses of variance (ANOVA) for the seven attention tasks. The independent variable was accuracy and the dependent variable was age group for every attention task. The result revealed that there was no significant difference among the three age groups in terms of alertness [*F*(2,359) = 0.53, *p* > 0.05, partial η^2^ = 0.003]. Furthermore, for attentional switching, sustained attention, spatial attention, and supervisory attention, we observed no differences between the 7- to 8-year-old group and the 9- to 10-year-old group. However, the accuracy of 11- to 12-year-old group was significantly higher than that of the other two groups [attentional switching, *F*(2,359) = 11.75, partial η^2^ = 0.10; sustained attention, *F*(2,359) = 7.36, partial η^2^ = 0.10; spatial attention, *F*(2,359) = 7.29, partial η^2^ = 0.10; supervisory attention, *F*(2,359) = 11.61, partial η^2^ = 0.10, *ps* < 0.01]. There were significant differences among the three age groups in focused attention [*F*(2,359) = 18.37, *p* < 0.001, partial η^2^ = 0.10] and divided attention [*F*(2,359) = 45.19, *p* < 0.001, partial η^2^ = 0.20]. Bonferroni analysis showed that the accuracy of the 7- to 8-year-old group was significantly lower than that of the two older groups, and that the accuracy of the 9- to 10-year-old group was significantly lower than that of the 11- to 12-year-old group.

**Table 2:** Accuracy in the seven tasks across the three groups.

Age group	Gender	Alertness	Focused	Divided	Switching	Sustained	Spatial	Supervisory
7–8 years	Boy (*n* = 56)	0.9963 (0.01)	0.9439 (0.08)	0.7487 (0.15)	0.9045 (0.07)	0.9086 (0.07)	0.9457 (0.07)	0.6316 (0.22)
	Girl (*n* = 48)	0.9968 (0.01)	0.9015 (0.14)	0.7054 (0.15)	0.9085 (0.08)	0.9283 (0.07)	0.9590 (0.03)	0.6308 (0.15)
	Total (*n* = 104)	0.9965 (0.01)	0.9243 (0.11)	0.7287 (0.15)	0.9063 (0.07)	0.9177 (0.07)	0.9518 (0.05)	0.6312 (0.19)
9–10 years	Boy (*n* = 77)	0.9975 (0.01)	0.9549 (0.06)	0.7904 (0.11)	0.8977 (0.10)	0.9260 (0.06)	0.9482 (0.06)	0.6426 (0.23)
	Girl (*n* = 65)	0.9994 (0.00)	0.9585 (0.07)	0.7930 (0.13)	0.9480 (0.05)	0.9406 (0.05)	0.9585 (0.04)	0.7071 (0.21)
	Total (*n* = 142)	0.9984 (0.01)	0.9565 (0.07)	0.7916 (0.12)	0.9207 (0.08)	0.9327 (0.06)	0.9529 (0.05)	0.6722 (0.22)
11–12 years	Boy (*n* = 62)	0.9998 (0.00)	0.9832 (0.03)	0.8860 (0.08)	0.9455 (0.05)	0.9429 (0.09)	0.9700 (0.03)	0.7540 (0.19)
	Girl (*n* = 57)	0.9949 (0.03)	0.9858 (0.03)	0.8790 (0.11)	0.9596 (0.07)	0.9611 (0.05)	0.9733 (0.02)	0.7583 (0.17)
	Total (*n* = 119)	0.9975 (0.02)	0.9845 (0.03)	0.8827 (0.09)	0.9523 (0.06)	0.9516 (0.07)	0.9716 (0.03)	0.7561 (0.18)

*SD in parentheses.*

We excluded all erroneous responses from our computation of reaction times. Previous studies that had adult participants chose reaction time for analysis. In order to compare with the results of adults, we chose reaction time. The means and standard deviations of the reaction times are presented in **Table 3**.

**Table 3:** Reaction times in the seven tasks across the three groups.

Age group	Gender	Alertness	Focused	Divided	Switching	Sustained	Spatial	Supervisory
7–8 years	Boy (*n* = 56)	414 (80)	666 (61)	978 (133)	1,632 (343)	388 (56)	925 (210)	44,004 (14,709)
	Girl (*n* = 48)	441 (85)	686 (63)	978 (108)	1,717 (385)	442 (72)	968 (214)	43,997 (11,237)
	Total (*n* = 104)	426 (83)	675 (63)	978 (121)	1,671 (364)	413 (69)	945 (212)	44,001 (13,159)
9–10 years	Boy (*n* = 77)	364 (84)	639 (68)	861 (102)	1,557 (407)	372 (63)	750 (207)	36,877 (13,150)
	Girl (*n* = 65)	369 (81)	618 (58)	866 (130)	1,415 (388)	387 (73)	781 (237)	36,916 (9,525)
	Total (*n* = 142)	367 (82)	629 (65)	863 (115)	1,493 (403)	379 (68)	764 (220)	36,895 (11,592)
11–12 years	Boy (*n* = 62)	324 (49)	602 (58)	764 (121)	1,214 (264)	357 (60)	596 (131)	30,692 (7,199)
	Girl (*n* = 57)	320 (62)	563 (72)	756 (95)	1,298 (323)	342 (55)	598 (140)	31,098 (6,022)
	Total (*n* = 119)	322 (55)	583 (67)	760 (109)	1,254 (296)	350 (58)	597 (135)	30,887 (6,636)

*SD in parentheses.*

### Correlations between Tasks

The correlation matrices of reaction times among the seven tasks in the three groups are shown in **Table 4**. Many of these correlations were significant.

**Table 4:** Correlations among reaction times in the seven tasks among the three age groups.

Age group		1	2	3	4	5	6	7
7–8 years	(1) Alertness RT	–						
	(2) Divided RT	0.399^∗∗^	–					
	(3) Sustained RT	0.418^∗∗^	0.390^∗∗^	–				
	(4) Switching RT	0.346^∗∗^	0.391^∗∗^	0.196^∗^	–			
	(5) Focused RT	0.338^∗∗^	0.377^∗∗^	0.516^∗∗^	0.216^∗^	–		
	(6) Spatial RT	0.444^∗∗^	0.569^∗∗^	0.321^∗∗^	0.472^∗∗^	0.386^∗∗^	–	
	(7) Supervisory RT	0.204^∗^	0.403^∗∗^	0.339^∗∗^	0.420^∗∗^	0.402^∗∗^	0.386^∗∗^	–
9–10 years	(1) Alertness RT	–						
	(2) Divided RT	0.359^∗∗^	–					
	(3) Sustained RT	0.518^∗∗^	0.450^∗∗^	–				
	(4) Switching RT	0.354^∗∗^	0.267^∗∗^	0.241^∗∗^	–			
	(5) Focused RT	0.538^∗∗^	0.487^∗∗^	0.564^∗∗^	0.301^∗∗^	–		
	(6) Spatial RT	0.473^∗∗^	0.456^∗∗^	0.546^∗∗^	0.564^∗∗^	0.450^∗∗^	–	
	(7) Supervisory RT	0.216^∗∗^	0.406^∗∗^	0.221^∗∗^	0.358^∗∗^	0.214^∗^	0.448^∗∗^	–
11–12 years	(1) Alertness RT	–						
	(2) Divided RT	0.411^∗∗^	–					
	(3) Sustained RT	0.308^∗∗^	0.258^∗∗^	–				
	(4) Switching RT	0.433^∗∗^	0.416^∗∗^	0.231^∗^	–			
	(5) Focused RT	0.343^∗∗^	0.309^∗∗^	0.298^∗∗^	0.180	–		
	(6) Spatial RT	0.409^∗∗^	0.428^∗∗^	0.245^∗∗^	0.624^∗∗^	0.329^∗∗^	–	
	(7) Supervisory RT	0.116	0.201^∗^	0.092	0.306^∗∗^	0.053	0.388^∗∗^	–

*^∗^*p* < 0.05, ^∗∗^*p* < 0.01.*

### Exploration of Attention Structure

To analyze the structure of attention in the three age groups, we employed the factor analysis method. The Kaiser–Meyer–Olkin indexes were 0.82, 0.82, and 0.79 for the 7- to 8-, 9- to 10-, and 11- to 12-year-old groups, respectively, indicating that the data were suitable for factor analysis. Factors were extracted if they had an eigenvalue of larger than 1. The results indicated that only one factor emerged in the 7- to 8-year-old group, which accounted for 46.86% of the total variance. Conversely, in the 9- to 10-year-old and 11- to 12-year-old groups, two factors emerged, accounting for 64.12% and 57.58% of the total variance, respectively (**Table 5**).

**Table 5:** Principal component analysis of attention structure in the three age groups.

	7–8 years	9–10 years		11–12 years	
	Factor 1	Factor 1	Factor 2	Factor 1	Factor 2
	Attention	Perceptual	Executive	Perceptual	Executive
		attention	attention	attention	attention
Alertness	**0.654**	**0.754**	0.194	**0.664**	0.325
Focused	**0.674**	**0.826**	0.140	**0.745**	0.021
Sustained	**0.659**	**0.825**	0.143	**0.670**	0.050
Divided	**0.751**	**0.562**	0.417	**0.534**	0.449
Spatial	**0.762**	0.519	**0.661**	0.372	**0.753**
Switching	**0.626**	0.201	**0.747**	0.318	**0.743**
Supervisory	**0.652**	0.063	**0.824**	-0.166	**0.776**

*The numbers in the table are the factor loadings of every task on each factor. The bolded operation in the table is to highlight that the corresponding attention tasks are included as one factor.*

As there was one factor in the 7- to 8-year-old group, we named the factor simply *attention*. In the 9- to 10-year-old and 11- to 12-year-old groups, Factor 1 comprised alertness, focused attention, divided attention, and sustained attention, while Factor 2 comprised attentional switching, spatial attention, and supervisory attention. Given that the attention measures in Factor 1 were more reliant on perceptual resources, the factor was named *perceptual attention*. Conversely, the measures in Factor 2 were more reliant on executive resources, so it was named *executive attention*.

## Discussion

Attention is one of the most important basic cognitive abilities in humans, and numerous studies have examined its development. Using factor analysis, we explored how the attention structure differed among children of different ages. The results showed that attention could not be divided into perceptual and executive attention among 7- to 8-year-olds, but it could be in 9- to 12-year-olds.

The findings for the 7- to 8-year-old children indicate that children’s attention at this age cannot be separated into two aspects, unlike in adults and the other two groups. Studies on executive function development are somewhat consistent with our findings, too. Klenberg et al. (2001) studied the attention and executive function of children aged 3–12 years and found that the developmental curves of these two cognitive abilities differed – in particular, attention developed earlier than did executive function. Duan (2009) later found that there was no stable executive function construct in 7- to 8-year-olds but a stable construct similar to the one observed in adults was found among 9-year-olds. These findings explain, to some degree, the lack of an adult-like attention structure among 7- to 8-year-olds.

Working memory is a key component of executive function. It is not strange that some studies could explain our results from the perspective of working memory. Developmental studies have shown adults’ working memory capacity tends to be larger and their executive function is better than children’s (Chevalier, 2015; Tsubomi and Watanabe, 2017). Given that a number of the attention tasks required the use of both working memory and executive function, it would naturally be easier for adults to complete them. Moreover, working memory capacity continues to develop throughout elementary school and does not even reach the adult level until 12 years old (Towse et al., 1998; Gathercole et al., 2004; Tsubomi and Watanabe, 2017). Thus, the performance of the 7- to 8-year-olds on the attention tasks was likely limited by their relatively small working memory capacity. Furthermore, as their working memory capacity increases, children are better able to process more complicated attention tasks that include the executive component.

From 9 years old onward, children’s attention can be divided into two factors. However, the attention types making up these two factors appear to differ from those in adults. In Moosbrugger et al. (2006) and Schweizer et al.’s (2005) studies, alertness, focused attention, sustained attention, and spatial attention were all classified as perceptual attention, whereas divided attention, attentional switching, and supervisory attention were classified as executive attention. Conversely, in our study, alertness, focused attention, sustained attention, and divided attention were classified as perceptual attention, whereas spatial attention, attentional switching, and supervisory attention were classified as executive attention. Thus, there were two main differences between children and adults: spatial attention and divided attention.

In adults, spatial attention was classified as perceptual attention (Schweizer et al., 2005; Moosbrugger et al., 2006), whereas among children, it was classified as executive attention. The spatial attention task required subjects to determine the position of a dot that might appear in four different positions (upper left, upper right, bottom left, and bottom right) and to make a quick response by pressing a corresponding key. We set four different corresponding keys in line with the four potential positions of the dot. As such, this task was likely very difficult for children, as to do well, they had to remember every dot and its corresponding key, and to choose the relevant key for each decision. Thus, this task would require considerable working memory capacity and executive function. Thus, it is reasonable that this spatial attention task would be classified as executive attention in children.

Polderman et al. (2009) selected 9-, 12-, and 18-year-old twins to clarify the shared genetic factors of inhibition and executive function, and found that inhibition and intelligence were related among the three age groups. This might be related to the level of development of children’s cerebral cortex. Kane and Engle (2002), in a review, suggested that there is a synchronicity between the development of attentional capacity and the cerebral cortex – in other words, cognitive maturity is based on physical maturity. Combining the results of the present study and those of this twin study, we might thus infer that although children aged 9–12 years possess the two attention components (perceptual and executive) that adults do, the actual processes still differ somewhat.

Divided attention, on the other hand, was classified as perceptual attention among children in the present study and executive attention among adults (Schweizer et al., 2005; Moosbrugger et al., 2006). This might be related to the time that participants were given for the task. In our study, children were asked to make a decision and press the corresponding key within 2,000 ms, after which the next trial began; thus, participants had little time to respond. Moreover, the divided attention task adopted a dual-task paradigm, meaning that participants would need to have sufficient resources to perform well on it. However, because of their lower maturity level in comparison to adults, children likely had fewer such resources. Under this condition, the participants might focus mainly on one task and thus would not make correct responses to the other tasks every time. Therefore, this might have led divided attention to be classified as perceptual attention alongside alertness, focused attention, and sustained attention.

Further evidence comes from the high cross-loadings for divided and spatial attention, especially the former. The loading of divided attention on the perceptual attention factor was smaller among the 9- to 10-year-olds than among the 11- to 12-year-olds, whereas the loading on executive attention was larger. This seems to suggest that divided attention was showing a developmental transition from perceptual to executive attention between the ages of 9–12 years. As for spatial attention, although we classified it as executive attention, it showed cross-loading on both factors. Again, however, the loadings on executive attention increased with age, which was not in accordance with our expectation (i.e., that the loadings on executive attention should be higher in the 9- to 10-year-old group than in the 11- to 12-year-old and loadings on perceptual attention should be lower). Despite this, the cross-loading on both factors persisted even among 11- to 12-year-olds. The cross-loadings indicate that these tasks had both perceptual and executive components. Taken together, our findings suggest that the structure of attention in older children, but not younger children, is similar to that of adults.

Besides the evidence affording by studies on executive function and working memory development, we attempted to provide an explanation from the perspective of the theory of visual attention (Bundesen, 1990) and executive control of theory of visual attention (Logan and Gordon, 2001) under the framework of Pascual–Leone’s Theory of Constructive Operators. Children aged 7–8 years can complete the whole visual attention process. However, they cannot complete the executive control of visual attention. The theory of Constructive Operators helps to clarify this trade-off between cognitive load and mental attentional capacity: children cannot solve a task if its cognitive load (number of relevant schemes that need activation) is above the child’s mental–attentional capacity (Pascual-Leone, 1970). This theory hypothesizes that mental–attentional capacity increases every other year after the age of 3 years, reaching a mental–attentional capacity of seven units at 15–16 years. And the consequence of this immature cognitive function is that their attention cannot be separated into two aspects. This is also the reason why divided attention was classified as perceptual attention among children.

Quite a few studies have revealed that children’s attention develops during the elementary school stage. From the perspective of attention structure, the present study offered empirical evidence of children’s attention development. The present study demonstrated that attention cannot be divided into two components at the 7–8-year-old stage and can be divided into perceptual and executive attention from the age of 9 years onward. However, the specific types of attention that each component includes are still different from that of adults, indicating that the attention function continues to develop after the age of 12 years.

## Author Contributions

TT and JS conceived and designed the experiments. TT performed the experiments, analyzed the data and wrote the main manuscript. All authors reviewed the manuscript.

## Conflict of Interest Statement

The authors declare that the research was conducted in the absence of any commercial or financial relationships that could be construed as a potential conflict of interest.
